# Identification of a Novel Variant of *ARHGAP29* in a Chinese Family with Nonsyndromic Cleft Lip and Palate

**DOI:** 10.1155/2020/8790531

**Published:** 2020-10-23

**Authors:** Jian-Xia Tang, Xiang-Shui Xiao, Kai Wang, Jie-Yuan Jin, Liang-Liang Fan, Rong Xiang

**Affiliations:** ^1^Department of Prosthodontics, Xiangya Stomatological Hospital & School of Stomatology, Central South University, Changsha, Hunan, China; ^2^Department of Oral and Maxillofacial Surgery, The Second Xiangya Hospital, Central South University, Changsha, Hunan, China; ^3^School of Life Sciences, Central South University, Changsha, China; ^4^Human Key Laboratory of Animal Models for Human Diseases, School of Life Sciences, Central South University, Changsha, China

## Abstract

**Background:**

Cleft lip with or without cleft palate (CL/P) is the most common facial birth defect, with a worldwide incidence of 1 in 700-1000 live births. CL/P can be divided into syndromic CL/P (SCL/P) and nonsyndromic CL/P (NSCL/P). Genetic factors are an important component to the etiology of NSCL/P. *ARHGAP29*, one of the NSCL/P disease-causing genes, mediates the cyclical regulation of small GTP binding proteins such as RhoA and plays an essential role in cellular shape, proliferation, and craniofacial development.

**Methods:**

The present study investigated a Chinese family with NSCL/P and explored potential pathogenic variants using whole-exome sequencing (WES). Variants were screened and filtered through bioinformatic analysis and prediction of variant pathogenicity. Cosegregation was subsequently conducted.

**Results:**

We identified a novel heterozygous missense variant of *ARHGAP29* (c.2615C > T, p.A872V) in a Chinese pedigree with NSCL/P.

**Conclusion:**

We detected the disease-causing variant in this NSCL/P family. Our identification expands the genetic spectrum of *ARHGAP29* and contributes to novel approaches to the genetic diagnosis and counseling of CL/P families.

## 1. Introduction

Cleft lip with or without cleft palate (CL/P) is one of the most prevalent human birth defects, with a worldwide incidence of 1 in 700-1000 live births [1]. The prevalence varies with ethnicity, sex, and cleft type [2]. According to whether patients have other organ malformations, CL/P is divided into syndromic CL/P (SCL/P) and nonsyndromic CL/P (NSCL/P). Although CL/P is found in more than 500 syndromes (including van der Woude syndrome 1 [OMIM_119300], ectrodactyly, ectodermal dysplasia, and cleft lip/palate syndrome 3 [OMIM_604292]), NSCLP preponderates in congenital facial cleft cases [3–5].

SCL/P follows Mendelian inheritance with disease-causing genes including *IRF6*, *TP63*, *TBX1*, and *SPECC1L* [5–8]. In contrast, NSCL/P is thought to have a complex etiology, with genetic factors acting in concert with environmental effects, which leads to variable phenotypes and incomplete penetrance [3, 9]. With the increasing availability of genome-wide association studies (GWAS) and whole-exome sequencing (WES), many genes have been identified as NSCL/P causative genes, including *CTNND1*, *PLEKHA5*, *PLEKHA7*, *CDH1*, and *ARHGAP29* [3, 10, 11].

*ARHGAP29* is located on 1p22.1 and encodes the Rho GTPase activating protein (GAP) 29. RAP1 is a small GTPase that regulates Rho GTPase signaling. ARHGAP29 and RAP1 effectors (RADIL and RASIP1) translocate to the plasma membrane, where they form a multiprotein complex mediating RAP1-induced inhibition of Rho signaling [12]. As a GTPase activator for the Rho-type GTPases by converting them to an inactive GDP-bound state, ARHGAP29 has a strong activity toward RhoA to suppress RhoA signaling and dampen ROCK and MYH9 activities in endothelial cells. Rho signaling plays an essential role in cellular shape, movement, cell-cell interactions, proliferation, and craniofacial development [13]. Hence, *ARHGAP29* mutations could lead to NSCL/P. In fact, *ARHGAP29* mutations have been shown to impede oral adhesions during orofacial development in mice.

In this study, we reported a NSCL/P family from Hunan province, China. We identified a novel missense variant of *ARHGAP29* (c.2615C > T, p.A872V) in the proband, which was inherited from his affected mother. To the best of our knowledge, this variant has not been reported in previous studies or presented in various single nucleotide polymorphism (SNP) databases.

## 2. Material and Methods

### 2.1. Patients and Subjects

The Review Board of Xiangya Stomatological Hospital of the Central South University approved this research (approval number 20190038). 51 CL/P patients were recruited, and 10 were selected to undergo WES according to family history and disease severity. Written informed consent was obtained from patients and their guardians, in which all subjects are consented to this study and the publication of the images. Blood was collected from the proband and related family members. Segregation analysis was performed in all family members based on the WES results.

### 2.2. Whole-Exome Sequencing

Genomic DNA was extracted with the DNeasy Blood and Tissue Kit (Qiagen, Valencia, Calif., USA). The Berry Genomics Co., Ltd. (Chengdu, China) provided the exome capture, high-throughput sequencing, and common variant filtering. The clustering of the index-coded samples was performed on the cBot Clster Generation System and Hiseq PE Cluster Kit (Illumina) according to the manufacturer's instructions. After cluster generation, the DNA libraries were sequenced on the Illumina Hiseq platform, and 150 bp paired-end reads were generated. After filtering the common variants (frequency ≥ 0.05) using the 1000 Genomes Project database (https://www.genome.gov/27528684/1000-genomes-project/), the Chinese Millionome Database (https://db.cngb.org/cmdb/), the Genome Aggregation database (http://gnomad.broadinstitule.org), and the Exome Aggregation Consortium database (http://exac.broadinstitute.org/), unique single-nucleotide polymorphisms (SNPs) were identified. Potential causative variants were screened by the list of genes related to NSCL/P (Table S1) and then predicted using bioinformatic programs including MutationTaster (http://www.mutationtaster.org/), Polyphen-2 (http://genetics.bwh.harvard.edu/pph2/), and SIFT (http://provean.jcvi.org/index.php). The analyses of gene function, inheritance pattern, and clinical phenotype were conducted using Online Mendelian Inheritance in Man (OMIM) (https://www.omim.org).

### 2.3. Cosegregation Analysis

Primer pairs were designed via DNASTAR. The primers sequences will be provided upon request (ARHGAP29 c.2615C > T f: CAGGGTAGTAGATCATGCAGAAG; ARHGAP29 c.2615C > T r: GGTGATAACAGAGGCTTTGGA). The target fragments were amplified via polymerase chain reaction (PCR) and analyzed using the ABI 3100 Genetic Analyzer (ABI, Foster City, CA).

## 3. Results

### 3.1. Clinical Features

**Figure 1:**
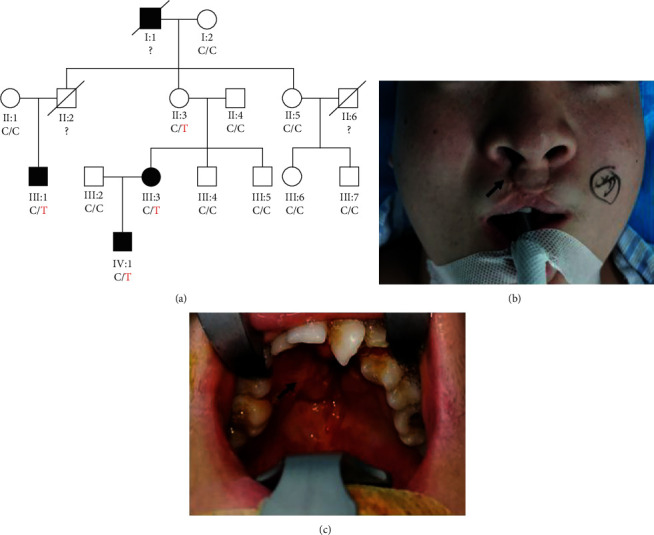
(a) Pedigree of the CL/P family with segregation analysis. The black symbols represent an affected member, and the arrow indicates the proband. Genotypes are identified by letters and slash, with red representing the variant. (b, c) The orofacial phenotypes of the proband. The proband has the scar due to the cleft lip repair (b), palate fistula, and altered dentition (c).

We collected 10 CL/P families to screen for mutations by WES and identified the genetic lesion for one family. This family was from the Hunan Province, China (Figure 1(a)). The proband (II : 1), a 16-year-old boy, was admitted to our hospital for a second palate fistula operation. The proband was diagnosed with cleft lip and palate at birth and had palate fistula and altered dentition without other organic abnormities (Figures 1(b) and 1(c)). The proband underwent lip repair surgery in the local hospital when he was 3 years old. His mother (III : 3), uncle (III : 1), and grandfather (I : 1) had CL/P. Other family members were unaffected.

### 3.2. Genetic Analysis

WES yielded 12.1 Gb of data with 98.22% coverage of the target region and 97.54% of the target covered over 10×. After a series of database analyses and filtering, 892 unique SNPs were detected in the proband. The variants were filtered by NSCL/P genes (Table S1), and a set of eight heterozygous variants in seven genes in the proband was identified (Table 1). By analyzing the bioinformatic prediction, inheritance pattern, OMIM clinical phenotypes, and American College of Medical Genetics classification [14] of these eight genes, we suspected one of the *ARGHAP29* variants (c.2615C>T, p.A872V) to be the causative variant in the family.

**Figure 2:**
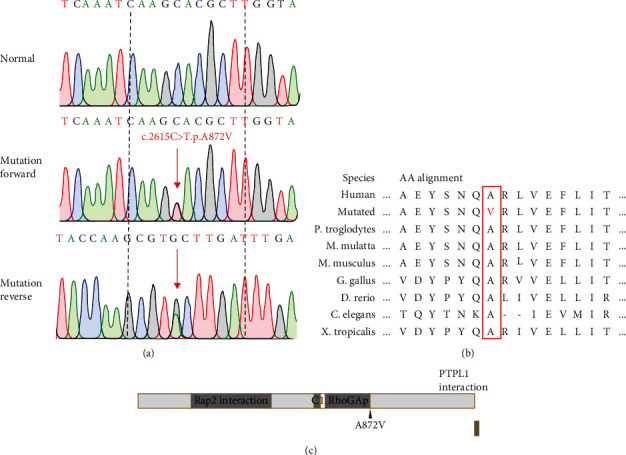
(a) The sequencing results of the *ARHGAP29* variant. Sequence chromatograms indicate the heterozygous variant (c.2615C > T, p.A872V) in the CL/P family. (b) The mutated site (A872V) is highly evolutionary conserved cross species. The red graphic represents a mutated amino acid, and the red box emphasizes cross species comparison. (c) A schematic diagram of the ARHGAP29 structure and the mutated AA site (A872V). Gray boxes represent the domains. “Rap2 interaction” indicates a coiled-coil region known to interact with Rap2; “C1” indicates a C1 domain; “RhoGAP” indicates a Rho GTPase domain; “PTPL1 interaction” indicates a small C-terminal region that interacts with PTPL1; and the black arrow represents the mutated AA site.

Sanger sequencing results indicated that the *ARHGAP29* variant (c.2615C>T, p.A872V) (Figure 2(a)) in the proband was inherited from his mother. Further sequencing in all subjects showed that this variant only existed in all affected subjects (III : 1, III : 3, and IV : 1) and II : 3. Additionally, the amino acid sequence alignment analysis suggested that the altered site was located in a highly evolutionarily conserved site (Figure 2(b)). Therefore, we considered the *ARHGAP29* variant (c.2615C>T, p.A872V) to be the main pathogenic factor in this family.

## 4. Discussion

CL/P is one of the most common developmental deformities with an incidence rate of 1.67‰ in China, of which approximately 70% cases are NSCL/P [15]. In NSCL/P cases, 80% are sporadic and 20% are familial cases [16]. Although NSCL/P is associated with various factors and not attributed to single etiologic mechanism, mutation screening in CL/P families could contribute to our understanding of genetic factor influence [17]. Pathogenic or likely pathogenic variants are identified in approximately 14% of multigenerational families with moderate to high penetrance [3]. In the present study, we reported a NSCL/P family across four generations with moderate penetrance. We confirmed presence of a heterozygous *ARHGAP29* variant (c.2615C > T, p.A872V) in all patients. Notably, II : 3 also harbored this variant, but was unaffected; however, incomplete penetrance is common in NSCL/P families. Regrettably, we did not test the variant of I : 1 and II : 2, who were considered to carry this base alteration, to further confirm the genotype-phenotype correlation.

CL/P results from facial morphogenesis and tissue fusion anomalies during embryonic development [13]. ARHGAP29 is a mediator of RhoA signaling that is related to cellular movement and proliferation in craniofacial development. Hence, ARHGAP29 defects are associated with CL/P. The structure of ARHGAP29 includes four domains: a coiled-coil region known to interact with Rap2, a C1 domain, the Rho GTPase domain, and a small C-terminal region that interacts with PTPL1 (Figure 2(c)) [18]. The alignment of the Rho GTPase domain covering 669^th^-881^st^ amino acids (AAs) in *ARHGAP29* is a highly conserved region that contains a catalytic residue and seven residues that compose the putative GTPase interaction site [9]. Ala at position 872 is one of putative GTPase interaction sites, and Val substitution at this site may thus affect the structure and function [9].

We summarized all known *ARHGAP29* mutations [9, 10, 18–21]. 19 *ARHGAP29* mutations have been reported in previous studies (Table 2). 18 mutations were identified in CL/P patients, and only one was detected in cleft palate (CP), suggesting *ARHGAP29* is highly associated with CL/P. In addition, incomplete penetrance of CL/P families with *ARHGAP29* mutations has been previously reported and confirmed in the present study. The moderate penetrance suggests that other factors play a role in CL/P occurrence, for example, environmental factors. Similarly, p.A832T and p.I845V in the Rho GTPase domain demonstrate the significant impact of this domain alteration for orofacial development [9].

## 5. Conclusions

In summary, the present study identified a novel heterozygous missense variant (c.2615C > T, p.A872V) of *ARHGAP29* in a Chinese family with CL/P. The identification expands the spectrum of known *ARHGAP29* mutations, further demonstrates the association of *ARHGAP29* and CL/P, and may contribute to novel approaches to the genetic diagnosis and counseling of CL/P families.

## Figures and Tables

**Table 1:** Variants identified by WES in combination with NSCL/P-related gene-filtering in the present family.

Gene	Variant	Mutation taster	PolyPhen-2	SIFT	1000G	ExAC	gnomAD	OMIM clinical phenotype	American College of Medical Genetics classification^∗^
*ARHGAP29*	c.2615C > T, p.A872V	D (1.000)	D (1.000)	D (0.001)	—	—	—	—	PM1, PM2, PP1
*ARHGAP29*	c.1252G > A, p.V418I	D (0.938)	B (0.241)	T (0.804)	0.00120	0.00149	0.00157		BS4, BP4
*ESRP2*	c.1610A > C, p.Y537S	D (1.000)	D (1.000)	D (0.000)	0.00399	0.00094	0.00109	—	PP3, BS4
*ABCA12*	c.1892G > A, p.R631Q	P (1.000)	B (0.050)	T (0.195)	0.00679	0.00525	0.00669	AR, ichthyosis, congenital, autosomal recessive 4A; AR ichthyosis, congenital, autosomal recessive 4B.	BS4, BP4
*BMP2*	c.393A > T, p.R131S	D (1.000)	D (0.575)	D (0.001)	0.00020	0.00058	0.00061	AD, brachydactyly, type A2; AD, short stature, facial dysmorphism, and skeletal anomalies with or without cardiac anomalies; AR HFE hemochromatosis, modifier of.	PP3, BS4
*GLI3*	c.3746G > A, p.C1249Y	P (1.000)	B (0.000)	T (1.000)	—	0.00006	0.00005	Somatic, hypothalamic hamartomas; AD, Greig cephalopolysyndactyly syndrome; AD, Pallister-Hall syndrome; AD, polydactyly, postaxial, types A1 and B; AD, polydactyly, preaxial, type IV.	BS4, BP4
*CHD7*	c.2496C > G, p.N832K	D (1.000)	D (0.900)	D (0.002)	—	—	0.00000	AD, CHARGE syndrome; AD, hypogonadotropic hypogonadism 5 with or without anosmia	PM2, PP3, BS4
*MYH9*	c.4872_4876delinsTCACG, p.I1626V	D (0.840)	B (0.041)	—	—	—	—	AD, deafness, autosomal dominant 17; AD, macrothrombocytopenia and granulocyte inclusions with or without nephritis or sensorineural hearing loss	PM2, BS4

D: disease causing; B: benign; T: tolerated; P: polymorphism; AR: autosomal recessive; AD: autosomal dominant. ^∗^pathogenic: PVS1>PS1>…>PS4>PM1-6>PP1-5; benign: BA1>BS1-4>BP1-7. PVS: pathogenic very strong; PS: pathogenic strong; PM: pathogenic moderate; PP: pathogenic supporting; BA: benign stand-alone; BS: benign strong; BP: benign supporting.

**Table 2:** Point mutations of *ARHGAP29* causing cleft in patients.

Mutation	Inheritance	Phenotypes	PMID
c.62_63delCT, p.S21YfsX20	AD	NSCLP	23008150
c.76A > G, p.T26A	AD	NSCL	23008150
c.94A > T, p.K32X	AD	NSCLP	25512736
c.137A > G, p.K46R	AD	NSCLP	23008150
c.698-1G > C	IP	NSCL/P	27350171
c.976A > T, p.K326X	AD	NSCLP	23008150
c.1475C > A, p.S492X	IP	NSCL/P	27350171
c.1576+1G > A	IP	NSCL/P	27350171
c.1654 T > C, p.S552P	AD	CP	28029220
c.1865C > T, p.T622M	AD	NSCLP	23008150
c.1939C > T, p.R647X	AD	CL/P	25704602
c.2109+1G > A	AD	NSCL/P	27350171
c.2367G > A, p.W789X	AD	CL/P	25704602
c.2494G > A, p.A832T	AD	NSCLP	23008150
c.2533A > G, p.I845V	AD	NSCLP	23008150
c.2615C > T, p.A872V	IP	NSCLP	
c.2738C > A, p.S913L	AD	CL	25081408
c.2864G > A, p.R955H	AD	NSCLP	23008150
c.3118G > T, p.G1040X	AD	CL/P	25704602
c.3425G > A, p.R1142Q	AD	NSCLP	23008150

Red word indicates the case in the present study. AD: autosomal dominant; IP: confirmed incomplete penetrance; NSCLP: nonsyndromic cleft lip and palate; NSCL: nonsyndromic cleft lip; NSCL/P: nonsyndromic cleft lip with or without cleft palate; CP: cleft palate; CL/P: cleft lip with or without cleft palate; CL: cleft lip.

## Data Availability

The datasets used and/or analyzed during the current study are available from the corresponding author upon reasonable request.

## Supplementary Materials

Supplementary Materials Supplementary Table S1. Causative/potential causative genes related to NSCL/P.
