# Supplementary material for: Identification of a Novel Variant of ARHGAP29 in a Chinese Family with Nonsyndromic Cleft Lip and Palate
Source: Biomed Res Int. 2020 Oct 23;2020:8790531. doi: 10.1155/2020/8790531 (PMC7603555; doi:10.1155/2020/8790531)
Supplement: Supplementary Materials — Supplementary Table S1. Causative/potential causative genes related to NSCL/P. [file 8790531.f1.docx]

**Supplementary Table S1.** Causative/potential causative genes related to NSCL/P

| *ABCA12* | *ACACB* | *ACSS2* | *AHDC1* | *AMELX* | *ARHGAP29* |
| --- | --- | --- | --- | --- | --- |
| *ARID5B* | *BHMT* | *BMP2* | *BMP4* | *BMPR1B* | *CDC45* |
| *CDC45* | *CDH1* | *CDON* | *CFAP57* | *CHD1* | *CHD7* |
| *COL11A1* | *COL11A2* | *COL2A1* | *COLEC11* | *CREBBP* | *CRISPLD2* |
| *CTNND1* | *CX43* | *DICER1* | *DLX4* | *DMGDH* | *DUSP22* |
| *ESR1* | *ESRP2* | *EYA1* | *FAM49A* | *FGF10* | *FGF8* |
| *FGFR1* | *FGFR2* | *FLNA* | *FOXE1* | *FZD6* | *GABA* |
| *GAD67* | *GADD54G* | *GJA1* | *GLI1* | *GLI2* | *GLI3* |
| *GRHL3* | *GSC* | *HYAL2* | *IRF6* | *KRT18* | *LINC00640* |
| *LRP6* | *LRP8* | *MAFB* | *MID1* | *MMP16* | *MSX1* |
| *MYC* | *MYH9* | *NECTIN1* | *NOG* | *NTN1* | *OTX2* |
| *PAX7* | *PAX9* | *PHYH* | *PLEKHA5* | *PLEKHA7* | *PTCH1* |
| *PVRL1* | *RAD54B* | *RPS26* | *SATB2* | *SCD5* | *SEMA3E* |
| *SIX1* | *SIX3* | *SIX5* | *SIX6* | *SLC32A1* | *SMO* |
| *SOX9* | *SPECC1L* | *SPRY1* | *SPRY2* | *SUMO1* | *TAF1B* |
| *TBX1* | *TBX22* | *TCOF1* | *TFAP2A* | *TGFBR1* | *TGIF* |
| *TMEM19* | *TP63* | *VAX1* | *VIAAT* | *WDR65* | *WNT9B* |
